# Fatigue testing of three peristernal median sternotomy closure techniques

**DOI:** 10.1186/1749-8090-3-52

**Published:** 2008-09-24

**Authors:** Cameron Wangsgard, David J Cohen, Lanny V Griffin

**Affiliations:** 1 California Polytechnic State University, Department of Biomedical Engineering, San Luis Obispo, CA 93407, USA; 2 Alamo Cardiothoracic Surgery Associates, 525 Oak Centre, Suite 270, San Antonio, TX 78258, USA

## Abstract

**Background:**

Failure of a sternotomy closure because of closure system fatigue is a complication that may result in dehiscence and put the individual at risk for serious complications. The purpose of this study was to assess the fatigue performance of three peristernal median sternotomy closure techniques (figure-of-eight stainless-steel wires, figure-of-eight stainless-steel cables, or Pectofix Dynamic Sternal Fixation [DSF] stainless-steel plates) in order to quantify the potential risk of fatigue failure of these devices when subject to cyclic loads in physiologically relevant loading directions.

**Study Design:**

All tests were conducted on polyurethane foam sternal models. A cardiothoracic surgeon divided each sternal model longitudinally and repaired it with a closure device. Tests were performed using a materials testing system that applied cyclic loading in a uniaxial direction until the test model catastrophically broke or data run-out occurred. For each loading direction (lateral distraction and longitudinal shear), five trials of each closure technique were tested. Life data and location of device failure (if present) were evaluated. Statistical analysis was performed using regression with life data allowed for correlation between life data and the various closure techniques to develop risk assessment curves for each device.

**Results:**

The data show that the figure-of-eight stainless-steel cable and the DSF plate systems are considerably less likely to fail under both lateral distraction and longitudinal shear cyclic loading conditions as compared to the figure-of-eight stainless-steel wire system. Moreover, the figure-of-eight stainless-steel cable system is the most resistant to failure, particularly for high cycle counts.

**Conclusion:**

This study in addition to Cohen and Griffin's earlier published biomechanical comparison of the ultimate strength of these same three closure techniques provide extensive experimental evidence regarding the mechanical differences among these three peristernal median sternotomy closure techniques. All data support the hypothesis that both the DSF plate system and the stainless-steel cable system offer important advantages over figure-of-eight wire closure techniques; although twisted wires are the weak-link in the systems we tested.

## Background

Any physical disruption or infection in the sternal region following median sternotomy closure results in compromised wound healing and the risk of additional complications. Although these issues are reported in only 0.3–5% of cases, the associated risks are severe, resulting in mortality between 14 and 47% of the time [1,2]. Moreover, studies have indicated that physical forces generated by coughing or patient movement can cause dehiscence, the separation of the sternal halves due to bone cutting, or closure device failure [3-5]. This separation can lead to life-threatening complications and is the most common complication of sternotomies, reported in 0.30–0.90% of patients [6-8].

In materials science, mechanical fatigue refers to a mode of material failure that occurs in structures subjected to fluctuating and recurring stresses, occurring even when stress levels are considerably less than the ultimate strength of the material under a static load [9]. Observing that forces generated in the thoracic cavity such as coughing can occur frequently over time, a successful sternal closure device must be able to survive cyclic loading from repetitive physiological forces. Additionally, current literature shows that complications associated with sternotomies often occur within the first two weeks after surgery, establishing the possibility that fatigue is a prevalent cause of closure device failure and consequent sternotomy complications [10]. Thus, fatigue testing may be critical to understanding the wear and cause of complications in various sternal closure devices.

There have been several studies of fatigue loading sternotomy closure devices in cadaveric sterna, in which the closure fails in less than 500 cycles due to the cutting of the wire into the sterna, which is certainly a clinically important common failure mode [11]. However, there appear to be no biomechanical studies that reliably demonstrate failure of the closure device by fatigue, which is also a clinically relevant failure mode [12]. Failure of a closure system is seen clinically in the common finding of broken sternal wires following sternal closure.

Due to the variability in bone density, sternal size, and thickness between both animal and human cadaver specimens, a foam model sternum was used order to minimize confounding variables, minimize subject variability and focus solely on the mechanical performance of the respective sternal closure devices. The polyurethane foam models used in this study simulate the mechanical properties observed in human cadaveric sterna and minimize variability among sternal specimens [13]. Moreover, unlike bone, polyurethane foam mechanical properties, such as stiffness and strength, has been shown to be unaffected by how fast the loads are applied [14]. Therefore, long-term fatigue testing with these models can occur at higher than physiologic rates – shortening test duration – and still reliably simulate fatigue performance *in-vivo*.

**Figure 1:**
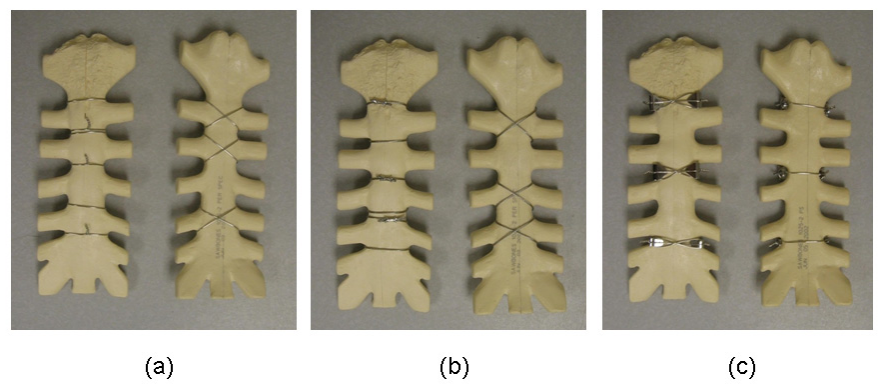
**Photographs of models closed using each test system.** (a = figure-of-eight stainless-steel wire; b = figure-of-eight stainless-steel cable; c = dynamic sternal fixation system sternal plates).

The purpose of this study was to investigate the mechanical fatigue performance of three peristernal median sternotomy closure techniques (figure-of-eight stainless-steel wires, figure-of-eight stainless-steel cables, or Pectofix Dynamic Sternal Fixation [DSF] stainless-steel plates) (Figure 1) in order to approximate the wear and potential failure of these devices when subject to physiologically relevant cyclic loads and loading directions. Common closure methods use a series of stainless-steel wires to secure the sternal halves created during the median sternotomy. The figure-of-eight is one of the most popular techniques utilizing this wire, and possesses theoretical advantages over interrupted simple circlage wires. The cable and DSF plate systems were both developed in response to the rare, but serious, complications that were observed with wire closure techniques. Both of these systems aim to reduce wear and fatigue-based failure by attenuating stresses on the sternum and regions of the closure device while additionally increasing the rigidity of the closure. Our hypothesis was that a sternotomy closure system that uses either stainless-steel cables or stainless-steel plates to distribute local stresses of the wires on the sternum will be less prone to fatigue-based failure *in-vivo *when compared to stainless steel wires. Avoiding re-operation due to device failure because of fatigue should facilitate bone healing as well as reduce the risk of postoperative complications.

## Methods and materials

The sternal models used in this study were fabricated using 20-lb/ft^3 ^rigid polyurethane foam (1025-2, Pacific Research Laboratories Inc, Vashon, Washington) and simulate the manubrium, sternal body, and xiphoid process of the sternum including approximately 2.5 cm of the surrounding ribs extending from the sternum. The sternal models were sawed in half longitudinally and were re-approximated by a board-certified cardiothoracic surgeon familiar with all three closure devices. The sterna were closed with either three peristernal figure-of-eight No. 5 stainless-steel wires (Ethicon Inc, Somerville, NJ), three figure-of-eight stainless-steel cables (Pioneer Surgical Technology, Marquette MI), or three pairs of stainless-steel plates (DSF Dynamic Sternal Fixation System, Pectofix Inc, South Plainfield, NJ).

Each reconstructed sternal model was set into a recessed fixture using a polyurethane resin (Roto-One Fast Cast, Goldenwest Manufacturing Inc, Cedar Ridge, CA) and secured transversely with cotter pins. The sternal models were then mounted to a materials testing system (Enduratec SmartTest SP, Bose Corporation, Minnetonka, MN) using custom fixtures to allow for displacement in one of two desired directions (lateral distraction or longitudinal shear). The selection of fatigue load parameters for each loading direction was based on published yield loads for these three sternotomy closure techniques as well as predicted distraction forces using the Casha *et al *mathematical model [5,15]:

(1) *T *= *rlP*.

**Figure 2:**
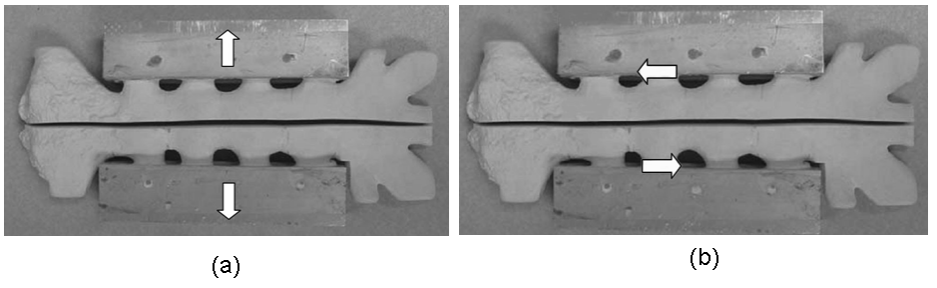
**Directions of load application on sternal models.** (a = lateral distraction; b = longitudinal shear).

Here, *T *is the distraction force in Newtons, *r *is the average radial distance of the chest, *l *is the average length of the sternum, and *P *is the pressure applied to the chest walls. Lateral distraction loading simulates breathing and coughing in a patient which cause the expansion of the thoracic cavity resulting in forces pulling the sternal halves away from each other (Figure 2). Fifteen models (5 of each closure technique) were tested in the lateral distraction phase subject to a cyclic load, sinusoidal in form, alternating between 25 N and 700 N, tested at 10 Hz.

Relevant geometry parameters published by Losanoff *et al *were used in the calculations using equation 1 [3]. The average radial distance of the chest (*r*) in our calculations was 0.15 m, the average length of the sternum (*l*) was 0.20 m, and the pressure applied to the chest walls (*P*) was 175 Pa. Locke *et al *examined respiratory pressures and associated rib cage mechanics in patients that had recently undergone a median sternotomy procedure [12]. In that study, maximum inspiratory pressures were reported: 61 mmHg one week after surgery; 59 mmHg three months following surgery; 74 mmHg for subjects with no history of a sternotomy. Losanoff *et al *reported a range of pressures within the lungs of 100–300 mmHg during coughing episodes [3]. Using Equation 1, resultant lateral forces for this range of thoracic pressures (59–300 mmHg) as reported by Locke and Losanoff were found to be 236–1200 N. This range is approximately 0.2 – 1.0 times the reported yield loads for these three sternotomy closure techniques in the lateral distraction loading mode in our previous study using this model [15].

Longitudinal shear simulates lateral flexion stretching, lying on one's side, or supporting the body on one vertically oriented arm (Figure 2). McGregor and coworkers reported that forces up to 325 N in longitudinal shear are physiologic [16]. Because the events that cause longitudinal shear occur in nearly equal frequencies between the left and right sides of the body, the models were subject to a sinusoidal force of ± 300 N resulting in the complete cycling of the sternal halves between one another. The peak value of 300 N is approximately 20 percent of the average ultimate load in the longitudinal shear direction using Cohen and Griffin's ultimate strength values for these same three sternal closure devices [15]. A cyclic wave period of 1 Hz was used for this loading mode in order to allow for complete displacement of the sternal halves, approximately 1–2 mm, and maintain proper load control. Fifteen models (5 of each closure technique) were tested in longitudinal shear.

After each test was completed, the number of cycles was recorded. For analysis purposes, if the sternal closure device did not fail by fatigue during testing, the number of cycles tested with the model was recorded and annotated as run-out data, which means that failure did not occur during testing, but presumably would at some time in the future if the test had continued.

Regression with life data was performed (Minitab 15.2, Minitab Inc, State College, PA) for both the lateral distraction test and the longitudinal shear test. This regression was performed for various probability distributions commonly utilized in accelerated testing: smallest extreme value, Weibull, exponential, normal, lognormal, logistic, and loglogistic. Run-out data were defined as right-censored and included in this analysis. The probability plot for standardized residuals in addition to an adjusted Anderson-Darling statistic was calculated for each distribution and the best fitting distribution was selected. Finally, using best fitting distribution, a table of survival probabilities was generated for the various models tested, from which relative risk curves were generated. The relationship of how closure technique affects fatigue life was also assessed using a post-hoc statistical analysis of the mean fatigue life (50% probability of survival) using Tukey's HSD. Statistically significant differences were reported when p < 0.05.

## Results

**Figure 3:**
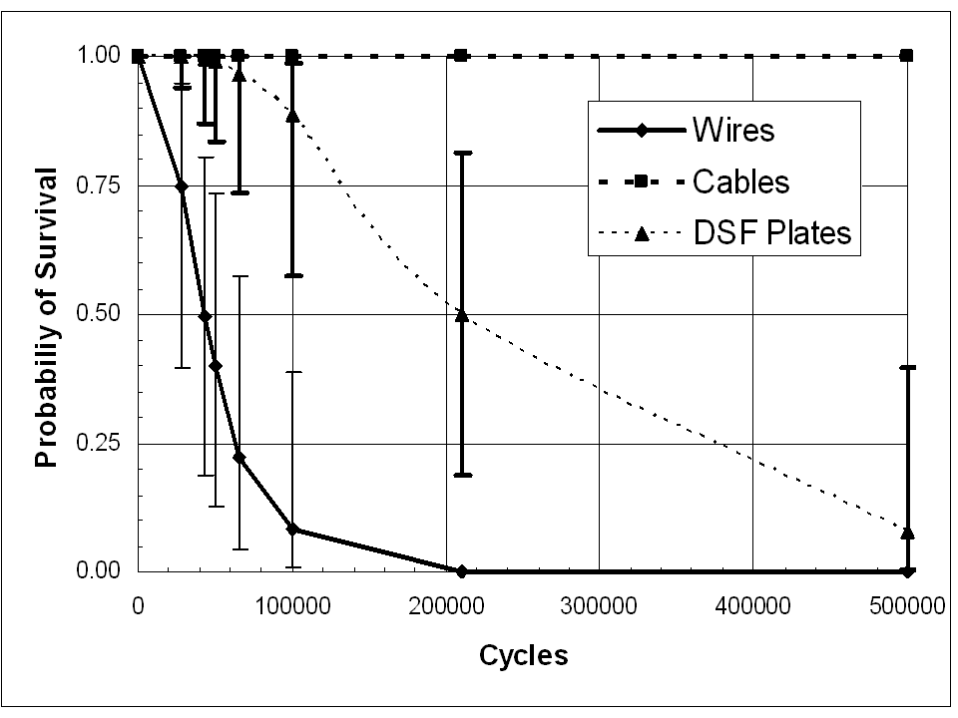
**Survival probability distributions for models subject to lateral distraction cyclic loading.** The 50% probability of survival (if it exists) is analogous to the mean fatigue life.

The probability of survival of the distraction fatigue tests demonstrates significant performance differences (Figure 3). The error bars associated with the curves do not appear for the cables because we were unable to obtain failure. One test we ran to 7.8 million cycles and stopped because the polyurethane sternum broke. The differences based on the 50 percent probability of survival among all constructs were found to be significantly different with the cables superior to the DSF, and the wires as the least reliable construct (Table 1).

**Table 1:** *Post-hoc *statistical analysis of the means (50% survival) for the fatigue tests.

**Distraction Mode**		
Construct	50% Survival	Std Err

Wires^a^	42866	11735
Cables^b^	1000000	
Plates^c^	209593	58459

**Longitudinal Shear**		

Construct	50% Survival	St Err

Wires^a^	730	205
Cables^b^	187855	115570
Plates^c^	46715	14384

Notes: Different lettered superscripts indicate the mean differences are statistically significant (p < 0.05). Comparisons are within the test groups distraction and shear.

**Figure 4:**
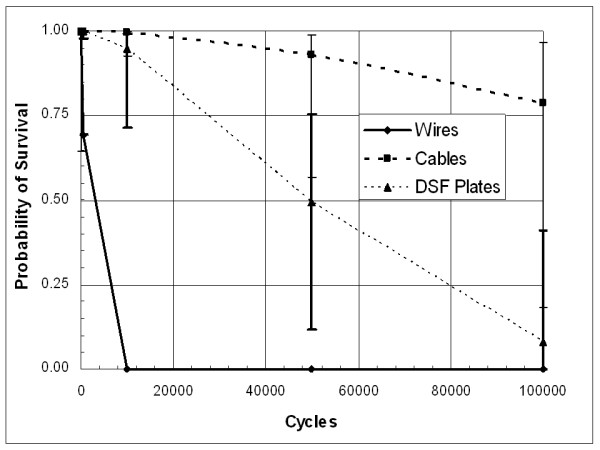
Survival probability distribution for models subject to longitudinal shear cyclic loading.

The survival probability curves for the longitudinal shear tests also demonstrate the same trends as the distraction tests (Figure 4). Here, the cables are still the most reliable construct; although there was one failure which allowed for an estimate of the error. As with the distraction tests, there are statistically significant reliability difference (Table 1).

## Discussion

The purpose of this study was to investigate the mechanical fatigue performance of three peristernal median sternotomy closure techniques with a view toward evaluating the relative risks of fatigue failure. Our hypothesis was that a sternotomy closure system that uses either stainless-steel cables or stainless-steel plates to distribute local stresses of the wires on the sternum will be less prone to fatigue-based failure *in-vivo *when compared to stainless steel wires.

The number of cycles for distraction used in this study is based upon the idea that 95% of bone healing occurs at 6 weeks (42 days). An estimate of *in-vivo *loading for lateral distraction at a rate of 3 coughs/min would be 181,440 cycles over 6 weeks. While these values may initially seem to over estimate the number of coughing events, it should be considered that a cough is rarely an isolated event. Furthermore, it is expected that chest pressures for some coughing events will be greater than those used to estimate our peak force. Since the relationship between fatigue life and stress is highly non-linear, high stresses produced by high forces will substantially lower the fatigue life of materials. Therefore, we believe that our values used provide a reasonable approximation for the longitudinal distraction loading.

### Figure-of-eight wires

**Figure 5:**
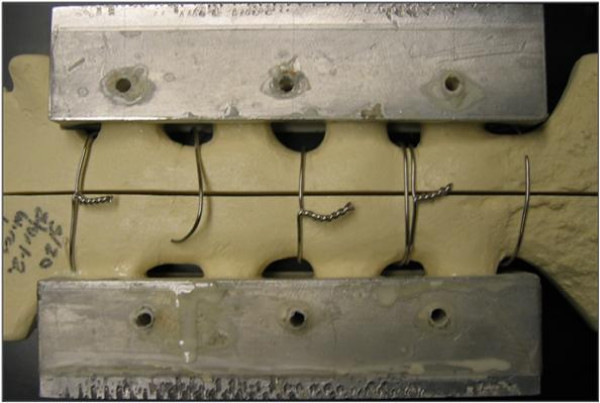
Typical failure of the figure-of-eight stainless steel wire device after longitudinal cyclic loading (anterior view).

All figure-of-eight stainless-steel wire closures failed during testing. Through visual observations, the failure of the figure-of-eight stainless steel wire closure devices occurred in one of two locations. For models subject to the lateral distraction test, constructs that used stainless steel wires failed at one or more sites where the wires were twisted due to the residual stress associated with significant plastic deformation of the wire. The longitudinal shear test led to fracture of the wire at one or more locations where the wire was bent around the intercostal space, where it traverses the anterior and posterior side of the sternum (Figure 5); this bending would also be a site of increased stress concentrations, resulting in compromised mechanical properties in the bent portion of the wire.

Under distraction loading, only 43% of figure-of-eight stainless-steel wire closure devices are predicted to survive for 50,000 cycles and by 100,000 cycles, nearly all these constructs will have failed (probability of survival = 8.3%). For longitudinal shear loads, only 69.4% of figure-of-eight stainless-steel wire closure devices are predicted to survive for 500 cycles. By 10,000 cycles, virtually all these devices fail (probability of survival < 0.001%).

### Figure-of-eight cables

**Figure 6:**
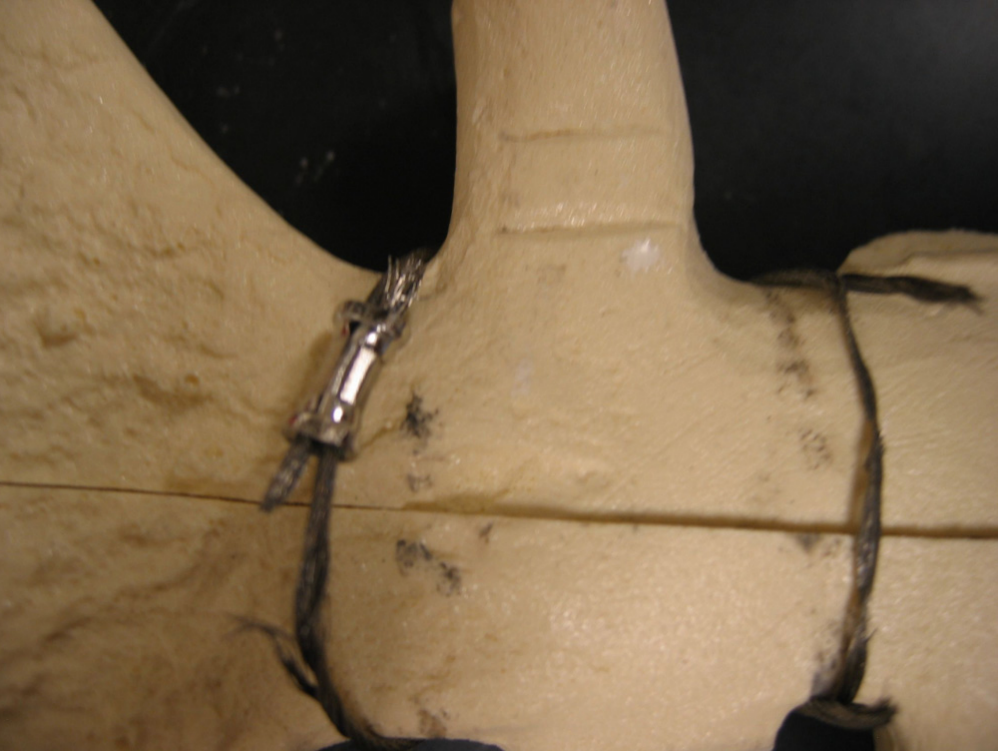
Site of failure in one model fixed with the figure-of-eight stainless steel cable that exhibited catastrophic failure during testing.

**Figure 7:**
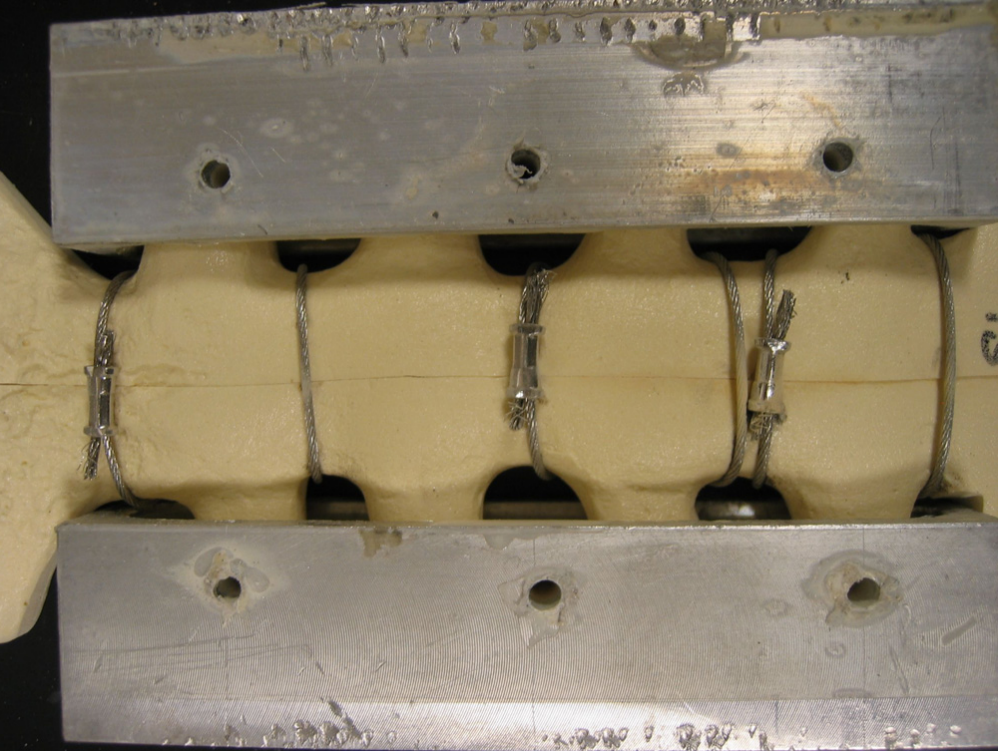
**Typical result of stainless-steel cable model after fatigue testing (anterior view).** No macroscopic failure was noticed following testing. Wear debris was observed from the foam model and the cable device.

For distraction, the probability of failure occurring in figure-of-eight stainless-steel cable closure failing at less than 500,000 cycles is estimated at less than 0.1 percent. For longitudinal shear, the probability of survival to 10,000 cycles (probability of survival = 99.5%) compared to virtually no possibility for the wires surviving to 10,000 cycles (probability of survival < 0.001%). Only one model fixed with the stainless-steel cable device failed during testing. This model failed when the cables frayed along the anterior side of the sternum (Figure 6). Debris from the polyurethane sternum models had built up on several of the cable devices, and most of the sternum models had areas covered with stainless-steel debris particles that had worn off the closure device during fatigue testing (Figure 7). None of the other systems exhibited polyurethane wear debris, and so there is some concern that the cable possibly may cut into the sternum by a sawing action.

### Dynamic sternal fixation plates

**Figure 8:**
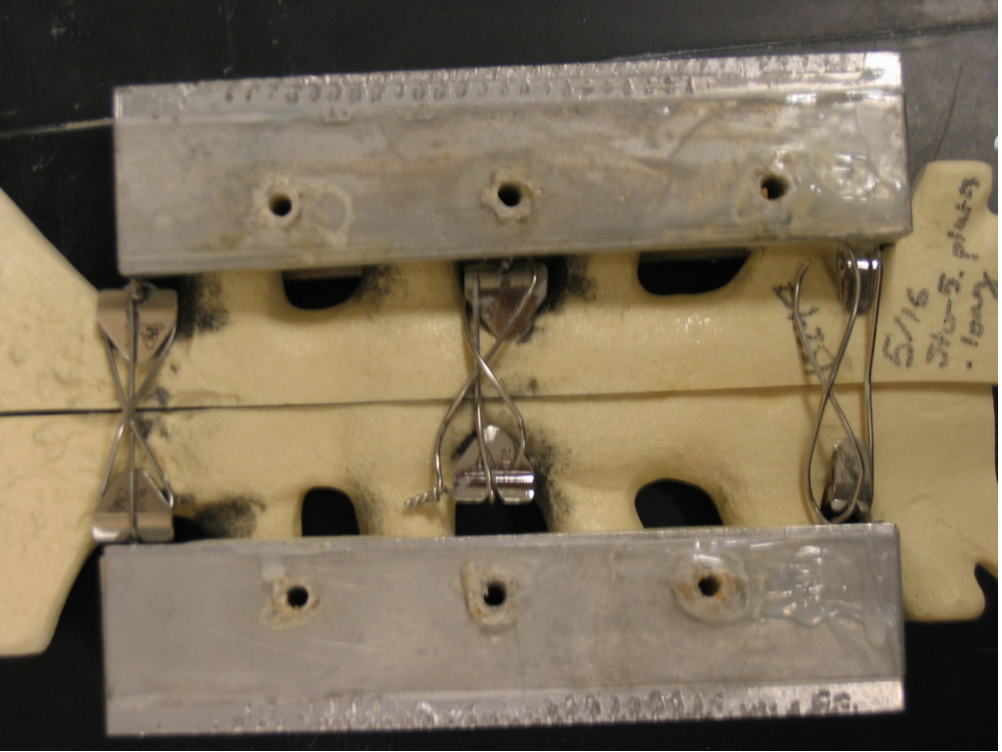
**Typical failure of DSF plate system (anterior view).** There was a substantially greater amount of wear debris compared with the other two closure techniques.

For distraction, the DSF plate system had a high probability of survival up to 100,000 cycles (88.7%), but catastrophic failure was almost certain to occur before 500,000 cycles (probability of survival = 7.8%). Several of the models fixed with the DSF plate system experienced catastrophic failure during testing. All failures of the DSF system were related to the stress concentration induced by twisting the stainless steel wires. There was also considerably more metallic wear debris compared with the other two closure techniques. Failure of the DSF also occurred at one or more notches in the plates where the wire is bent to pass between the anterior and posterior surfaces of the sternum (Figure 8). Additionally, the models rigidity decreased with time as evidenced by an increase in per-cycle peak-to-peak displacement; however, 40% of these models did not experience any catastrophic failure during testing. This cannot be said of the wires, which all failed.

### Comments

The present results demonstrate that the cables are superior to the DSF and then wires. The conclusions of this study are different from a previous biomechanical study on the same three constructs where it was reported that the DSF plates were favored over the cables. In that study, however, other parameters, such as stiffness, were considered which are thought better promote better healing by stabilizing the sternotomy closure; whereas the present study examines the durability under repetitive loads [15]. Adding additional wires to a figure of eight closure will improve the fatigue life, yet the sternal loading is complex and demanding, thus the materials will still fail with time. Therefore, the differences between studies are important since the both rigidity and durability are necessary for proper healing.

Most fatigue studies only focus on the notion of wires cutting into the sternum which leads to failure. It is extremely important to realize that while the repeated loads may cause the system to damage the sternum, the closure system itself is made of materials that will fail under repetitive loading. The value of this study is that we have shown clearly that the durability must also be considered when selecting a closure system.

The present study is *biomechanical *in nature, and each loading mode is considered separately. In an *in-vivo *situation, all loading occurs simultaneously. This suggests that the fatigue life *in-vivo *would occur in fewer cycles than predicted for either individual loading since the residual strength of the system is affected by each load cycle. That being said, the results of this study clearly demonstrate consistency among the system *ratings *between loading method and therefore, it is not expected that the ratings would change.

Furthermore, the testing we report on was done in air, without the additional environmental concerns, such as corrosive (saline) bodily fluids, that are known to shorten fatigue life [17,18]. Stainless steel alloys are known to be particularly susceptible to such environmental attacks. Therefore, while these fatigue lives may seem very long, it is quite possible that wires, cables, or other hardware associated with sternotomy closure may have the fatigue life shortened by at least a factor of 10, which then accounts for why there are reports of broken wires in the literature as well as the wires cutting into the sternum, leading to dehiscence [2,6,17-20]. Additionally, even if the closure method does not fail by fatigue, the debris generated by fatigue process could lead to metallosis or other foreign body reactions that can produce complications [21-23].

One of the most significant aspects of this study is that it provides a reliable means to evaluate the potential durability of a sternal closure system under fatigue. This information, when coupled with cadaveric studies, can provide valuable insight into how the system may affect the clinical outcomes. Obtaining long-term closure system durability information using cadavers would be more challenging since the test would span several days, and preserving the tissue from decomposition over the test duration would be difficult.

## Conclusion

1. The complicated combined loading induced by breathing, coughing, and body movement of a patient can all lead to failure of sternal closure devices by fatigue [3-5]. While this study examined each loading mode independently, the results should not differ for combined loading, except that the fatigue life is expected to be much shorter than the longitudinal shear tests due to the complex interaction of loading.

2. Adding additional wires or cables may improve the length of the fatigue life, but the weak link in most closure systems is the stainless steel wire. Therefore, any system that uses wires is still more likely to fail than a system that does not use them. Also, the use of more wires will not have a significant impact on the stress concentration induced on the sternum – which is a factor we did not evaluate. Considering this, any benefit that is derived by the addition of hardware to wires (e.g. the DSF), such as improved stiffness, should be considered carefully since the wires are still likely to fail, shifting loads to the remaining construct which may precipitate failure and lead to dehiscence.

3. As compared to the stainless-steel wire and cable systems, cyclic loading of the DSF plate system resulted in considerably more stainless-steel wear debris. Although the effects of metallic debris have not been reported on in the literature with sternotomy closure, current literature does report that metallic debris like this could lead to metal toxicity or corrosion assisted fatigue [17,20]. This study, in addition to Cohen and Griffin's published biomechanical comparison examining ultimate stress to failure testing of the same three closure techniques, provides extensive experimental evidence regarding the mechanical differences among these three peristernal median sternotomy closure techniques. All data supports the hypothesis that the cables are the best fixation method of these three techniques and that eliminating wires altogether may lead to an improved clinical outcome.

## Competing interests

The authors declare that they have no competing interests.

## Authors' contributions

CW carried out the majority of the testing, the statistical analysis, and manuscript preparation. DJW assisted in the study design, performed the closures for all the sterna used, and provided editorial feedback on the manuscript. LVG assisted in the study design, testing, statistical analysis, as well as manuscript preparation and revision. All the authors have read and approved the final manuscript.

## References

[B1] Satta J, Lahtinen J, Raisanen L, Salmela E, Juvonen T (1998). Options for the management of poststernotomy mediastinitis. Scand Cardiovasc J.

[B2] El Oakley RM, Wright JE (1996). Postoperative mediastinitis: classification and management. Ann Thorac Surg.

[B3] Losanoff JE, Collier AD, Wagner-Mann CC, Richman BW, Huff H, Hsieh F, Diaz-Arias A, Jones JW (2004). Biomechanical comparison of median sternotomy closures. Ann Thorac Surg.

[B4] Bruhin R, Stock UA, Drucker JP, Azhari T, Wippermann J, Albes JM, Hintze D, Eckardt S, Konke C, Wahlers T (2005). Numerical simulation techniques to study the structural response of the human chest following median sternotomy. Ann Thorac Surg.

[B5] Casha AR, Yang L, Kay PH, Saleh M, Cooper GJ (1999). A biomechanical study of median sternotomy closure techniques. Eur J Cardiothorac Surg.

[B6] Hehrlein FW, Herrmann H, Kraus J (1972). Complications of median sternotomy in cardiovascular surgery. J Cardiovasc Surg (Torino).

[B7] Stoney WS, Alford WC, Burrus GR, Frist RA, Thomas CS (1978). Median sternotomy dehiscence. Ann Thorac Surg.

[B8] Spencer K (2003). Developoment of a sternum closure fatigue test from an ultmate strength test method.

[B9] Callister WD (2003). Materials Science and Engineering: An Introduction.

[B10] Wilkinson GA, Clarke DB (1988). Median sternotomy dehiscence: a modified wire suture closure technique. Eur J Cardiothorac Surg.

[B11] Casha AR, Gauci M, Yang L, Saleh M, Kay PH, Cooper GJ (2001). Fatigue testing median sternotomy closures. Eur J Cardiothorac Surg.

[B12] Locke TJ, Griffiths TL, Mould H, Gibson GJ (1990). Rib cage mechanics after median sternotomy. Thorax.

[B13] Trumble DR, McGregor WE, Magovern JA (2002). Validation of a bone analog model for studies of sternal closure. Ann Thorac Surg.

[B14] McNee C (2004). A design analysis for fatigue testing sternomy closure techniques. Materials Engineering.

[B15] Cohen DJ, Griffin LV (2002). A biomechanical comparison of three sternotomy closure techniques. Ann Thorac Surg.

[B16] McGregor WE, Trumble DR, Magovern JA (1999). Mechanical analysis of midline sternotomy wound closure. J Thorac Cardiovasc Surg.

[B17] Shih CC, Shih CM, Su YY, Lin SJ (2004). Potential risk of sternal wires. Eur J Cardiothorac Surg.

[B18] Shih CM, Su YY, Lin SJ, Shih CC (2005). Failure analysis of explanted sternal wires. Biomaterials.

[B19] Harjula A, Jarvinen A (1983). Postoperative median sternotomy dehiscence. Scand J Thorac Cardiovasc Surg.

[B20] Jacobs JJ, Gilbert JL, Urban RM (1998). Corrosion of metal orthopaedic implants. J Bone Joint Surg Am.

[B21] Pearle AD, Scanzello CR, George S, Mandl LA, DiCarlo EF, Peterson M, Sculco TP, Crow MK (2007). Elevated high-sensitivity C-reactive protein levels are associated with local inflammatory findings in patients with osteoarthritis. Osteoarthritis Cartilage.

[B22] Pearle AD, Crow MK, Rakshit DS, Wohlgemuth J, Nestor BJ (2007). Distinct inflammatory gene pathways induced by particles. Clin Orthop Relat Res.

[B23] Nazzal A, Lozano-Calderon S, Jupiter JB, Rosenzweig JS, Randolph MA, Lee SG (2006). A histologic analysis of the effects of stainless steel and titanium implants adjacent to tendons: an experimental rabbit study. J Hand Surg [Am].

